# Enhancing prostate cancer detection: The role of b-value and apparent diffusion coefficient in DWI

**DOI:** 10.12688/f1000research.161128.3

**Published:** 2025-09-10

**Authors:** Brian Goveas, Winniecia Dkhar, Rajagopal Kadavigere, Kaushik Nayak, Abhimanyu Pradhan, Anand Venugopal, Praveen Shastry, Neil Barnes Abraham

**Affiliations:** 1 Medical Imaging Technology, Manipal College of Health Professions, Manipal Academy of Higher Education, Manipal, Karnataka, 576104, India; 2 Radiodiagnosis and Imaging, Katsurba Medical College, Manipal Academy of Higher Education, Manipal, Karnataka, 576104, India; 3 Radiodiagnosis, Manipal HealthMap, Manipal, Karnataka, 576104, India

**Keywords:** Prostate Cancer, Magnetic Resonance Imaging, Diffusion Weighted Imaging

## Abstract

**Background:**

Magnetic Resonance Imaging (MRI) is a highly effective tool for the detection of prostate cancer (PCa). Diffusion-weighted MRI (DW-MRI) is a sensitive technique that depends on the b value and apparent diffusion coefficient (ADC) value for the diagnosis of PCa. The main objective of this study was to determine the optimal b-value and apparent diffusion coefficient (ADC) value in DW-MRI for the diagnosis of prostate cancer.

**Methods:**

A prospective study including 26 male participants were conducted. MRI examinations were performed with T2 fat saturation sequences, and Diffusion weighted imaging (DWI) sequences with b-values (800, 1000, 1500, and 2000 mm ^2^/s) were used, and the corresponding ADC maps were calculated. Qualitative and quantitative analyses were conducted.

**Results:**

According to the present study, a b-value of 0,1500 mm ^2^/s exhibited the highest Signal-to-Noise Ratio (SNR), Signal Intensity Ratio (SIR), and Contrast-to-Noise Ratio (CNR). Area Under the Curve (AUC) for 0,1500 mm ^2^/s was 0.80, indicating a high diagnostic accuracy for prostate cancer.

**Conclusion:**

DWI with a b-value of 1500 mm ^2^/s provides good diagnostic accuracy for differential diagnosis of prostate lesions. DWI is a crucial sequence in multiparametric MRI of the prostate and offers detailed information that enhances the accuracy of prostate cancer diagnosis and management.

## Introduction

Prostate cancer (PCa) is a significant global health concern and the second-most common cancer among men in several regions of India. ^1^ MRI is a highly effective tool for the detection, treatment planning, and follow-up of prostate cancer (PCa), but its acceptance is not universal. Diffusion-weighted MRI (DW-MRI) can determine the distribution of water in tissues as well as the extracellular space and cell density within the tissues. ^2^ Diffusion-weighted imaging is sensitive to the movement of water molecules at the diffusion scale, where it focuses on the Brownian movement of water molecules and has a three-dimensional process that quantifies the diffusion index. This index reflects the apparent mean diffusivity, commonly referred to as the Apparent Diffusion Coefficient (ADC), which estimates the extent of diffusion along three orthogonal directions. ^3^ The sensitivity of this sequences depends upon the factor which is known as b-value. Diffusion weighted imaging (DWI) sequence is sensitive to prostate lesion detection, which can be adjusted by manipulating an extrinsic parameter known as the b-value. Higher b-values produce a stronger diffusion-weighted signal but result in a lower signal-to-noise ratio (SNR), whereas lower b-values are influenced by perfusion, which affects the sensitivity of diffusion sequences. ^4^ Despite its well-established diagnostic status in oncology, DWI presents challenges in its clinical application for evaluating prostate cancer owing to technical limitations and a lack of standardized protocols. This technique utilizes Echo Planar Imaging (EPI), which allows the rapid acquisition of images without the need for contrast agents. ^5, 6^ DWI has been used for prostate imaging in several previous studies; however, its sensitivity and specificity have varied, which is likely the result of variations in technical parameters, such as the selection of b values, strength of the magnetic gradient, and methods for calculating ADC values in the region of interest. ^7, 8^ The current literature lacks research on the optimal b-values and ADC values for prostate cancer screening and diagnosis. Establishing a single optimal b-value is crucial for reducing motion artifacts and time. Diffusion-weighted imaging (DWI) is particularly valuable for patients who are unable to undergo contrast-enhanced studies and may enhance the positive predictive value (PPV) for detecting prostate lesions. Thus, further investigations are essential to establish a standardized protocol. The main objective of the present study was to determine the diagnostic accuracy of the optimal b-value and ADC value of DWI for the detection of prostate cancer.

## Methods

Ethical approval for this prospective study was obtained from the Institutional Ethics Committee (IEC 126/2023) of Kasturba Hospital, Manipal, India, for data collection, on 4/05/2023. All participants were fully informed about the study’s objectives and procedures. Written informed consent was obtained in compliance with the ethical principles set forth in the Declaration of Helsinki. This study was registered in the Clinical Trial Registry of India (CTRI).

### Subjects

The study included 26 male subjects age group–35-75 years (mean±SD, 58±2 years) who had a confirmed diagnosis of prostate cancer on ultrasonography and were then recruited for MRI. Subjects who had undergone prostate surgery, chemotherapy, or radiotherapy were excluded.

### MRI techniques

MRI examinations were performed with a Philips Achieva© 1.5Tesla MRI - (Philips, Netherlands) and United Imaging uMR ^®^780uCS 3.0 Tesla - Tesla MRI (Shanghai United Imaging, China). A 12-channel pelvic phased-array coil is used. The examination protocol consists of a conventional pulse sequence - Axial T2W (Philips-Repetition Time (TR)/Echo Time (TE): 2494/100 ms, slice thickness: 3.5 mm, matrix, 224 × 199; Number of Excitations (NEX), 2; United-TR/TE, 4800/115 ms; slice thickness, 3 mm; matrix, 224 × 199; NEX, 2) for lesion localization and lesion size measurement, and DW sequence (Philips TR/TE: 5193/72 ms, flip angle: 90°, slice thickness: 4 mm, matrix: 108 × 86, signal averages: 1.011, United-TR/TE: 4520/73 ms, flip angle: 90°, slice thickness: 4 mm, matrix: 108 × 86, signal averages: 1.011) with a combination of four b-values (b- 800,1000,1500 and 2000 mm ^2^/s). The diffusion series was then registered before generating the corresponding ADC maps for each b-value ( Table 1).

**Table 1.** MRI sequence parameters for Philips Achieva 1.5T and United Imaging uMR 3.0T scanners.

Parameter	Philips 1.5T – DWI	Philips 1.5T – T2 FS	United Imaging 3T – DWI	United Imaging 3T – T2 FS
Slice Thickness	4 mm	3.5 mm	3 mm	3 mm
TR (ms)	5193	2494	4520	4800
TE (ms)	72	100	73	115
b-values (s/mm ^2^)	0, 500, 1000, 1500, 2000	–	0, 50, 800, 1500, 2000	–
Matrix	108 × 86	224 × 199	108 × 86	224 × 199
Fast Imaging Mode	EPI	–	EPI	–
Signal Average/NEX	1.011	2	1.011	2
DWI Directions	15	–	15	–
Flip Angle	90°	90°	90°	90°
Fat Suppression	SPIR	SPIR	SPIR	SPIR

### Image analysis

Qualitative and quantitative approaches were integrated in this study to provide a comprehensive assessment of image analysis. Qualitative analysis can provide context and insight, whereas quantitative analysis can offer precise and reproducible measurements. Qualitative image analysis was performed in the T2 FS sequence for localization and measurement of the lesion size in its maximum dimension in centimetres. The two radiologists, each with more than 10 years of experience, were blinded and analysed the DWI images at all different b-values. The image quality was assessed using a 5-point Likert scale, in which 1 represents unacceptable image quality, 2 = suboptimal, 3 = average, 4 = acceptable, and 5 = excellent, based on subjective SNR. ^9^

Quantitative analysis was conducted by measuring the signal intensity of the lesion, normal glandular tissue, and background noise, by placing the ROI on the acquired images. The SI values were used to compute the signal to intensity ratio (SIR) using the formula SIR= signal intensity of lesion/signal intensity of background noise, and contrast to noise ratio (CNR) using the formula signal intensity of lesion – signal intensity of tissue/standard deviation of background noise. The ADC values of the prostate lesions were calculated by drawing three ROIs within the lesion or tumor and three within the normal tissue; these values were averaged for each b-value across the ROIs. Histopathology reports were collected for all patients as part of the investigation.

### Statistical analysis

Data analysis was conducted using Statistical Package for Social Science (SPSS) version 16.0. ^10^ The inter-rate reliability of qualitative items (i.e., image quality) was determined using the kappa statistic. ^11^ Descriptive statistics were analyzed to determine the Mean and Standard Deviation of the ADC values of the prostate lesions. To determine the ADC cut-off value, receiver operating characteristic (ROC) curves were used, and the area under the curve (AUC) was used to calculate the sensitivities, specificities, and positive predictive values (PPV) of the multiple b values to determine the threshold ADC values. Youden’s index (J) was used to evaluate the diagnostic performance level (optimal b value) for the detection and differential diagnosis of diseases.

## Results

A total of 26 subjects were included in this study, 20 of whom had malignant lesions and 6 of which were benign lesions. To evaluate the image quality of DWI with different b values, both subjective and quantitative evaluations were conducted. Table 2 illustrates the subjective SNR based on the differences between the signal intensity in the region of interest and background tissue. The Kappa values for b value of 800, 1000, 1500, and 2000 mm ^2^/s were 0.77, 0.75, 0.64, and 0.64, respectively, which indicates moderate agreement across all b-values. To distinguish lesions from normal tissue, Kappa values varied from 0.78, 0.75, 0.77, and 0.79 for b-values of 800,1000,1500, and 2000, respectively. In the zone of the prostate, b = 1500 mm ^2^/s exhibited a higher inter reading agreement with a Kappa value of 0.79 compared with other b values. In addition, it was noted that the Geometric Distortion for b values 800 & 2000 mm ^2^/s had a higher inter reading agreement, with Kappa values of 0.9 and 0.93 as compared to the other b values. A quantitative analysis of the SIR, SNR, and CNR of prostate lesions from the DWI sequence is presented in Table 3. Compared to other b-values, the b-value of 1500 mm ^2^/s showed excellent signal intensity with minimal noise and excellent contrast differentiation between the lesions and normal tissue. Table 4 presents a quantitative analysis of the ADC values for prostatic lesions and normal tissues, including the b-values (800,1000,1500,2000 mm ^2^/s). The mean ADC values for normal tissues were 1.402±0.20 mm ^2^/s, 1.606±0.18 mm ^2^/s, 1.416±0.15 mm ^2^/s, and 1.25±0.20 mm ^2^/s for b-values of 800, 1000, 1500, and 2000 mm ^2^/s, respectively. It was noted that the mean ADC values for benign lesions were 0.708 ± 0.14 mm ^2^/s, 0.839 ± 0.15 mm ^2^/s, 0.665 ± 0.15 mm ^2^/s, and 0.59 ± 0.19 mm ^2^/s for the corresponding b-values. In a similar manner, the mean ADC values of malignant lesions were 0.524 ± 0.03 mm ^2^/s, 0.79 ± 0.014 mm ^2^/s, 0.52 ± 0.16 mm ^2^/s, and 0.48 ± 0.09 mm ^2^/s, respectively. Therefore, the ADC value at b-1500 mm ^2^/s was statistically significant for differentiating benign from malignant lesions. In this analysis, it was found that the mean ADC values decreased as the b-value increased, with malignant lesions exhibiting consistently lower ADC values. For a b-value of 800 mm ^2^/s, the ADC cut-off threshold value was 0.481 × 10 ^-3^ mm ^2^/s which yielded 90.9 sensitivity and 83.3%; for a b-value of 1000 mm ^2^/s, the ADC cut-off threshold value was 0.510 × 10 ^-3^ mm ^2^/s with 90.9% sensitivity and 79% specificity; for a b-value of 1500 mm ^2^/s, the ADC cut-off threshold value was 0.389 × 10 ^-3^ mm ^2^/s with 94% sensitivity and 87% specificity; and for a b-value of 2000 mm ^2^/s the ADC cut-off threshold value was 0.351 × 10 ^-3^ mm ^2^/s with 77% sensitivity and 88% specificity.

**Table 2.** The Kappa value of the subjective assessment of prostate lesions on a DWI.

b value (mm ^2^/s)	Criteria 1 Subjective SNR	Criteria 2 Lesion V/s tissue differentiation	Criteria 3 Zonal Anatomy	Criteria 4 Geometric Distortion
0,800	0.77	0.78	0.77	0.9
0,1000	0.75	0.75	0.74	0.81
0,1500	0.64	0.77	0.79	0.74
0,2000	0.64	0.79	0.78	0.93

**Table 3.** Quantitative assessment of Signal Intensity Ratio (SIR), Signal to Noise Ratio (SNR) and Contrast to Noise Ratio (CNR) of the prostate lesion on diffusion weighted images with respect to multiple b-values.

b value (mm ^2^/s)	Image Criteria’s		
	Signal Intensity Ratio	Signal to Noise Ratio	Contrast to Noise Ratio
0,800	1.86±0.540	6.31±2.251	87.04±70.778
0,1000	1.63±0.423	4.24±1.632	84.75±91.966
0,1500	1.51±0.630	6.99±2.749	117.43±60.019
0,2000	2.46±0.763	5.19±2.891	93.05±70.279

**Table 4.** Mean and Range of ADC values of Benign, Malignant and Normal Prostate tissues at multiple b value in MR Diffusion Weighted Imaging.

b value (mm ^2^/s)	Prostate Tissue	ADC Mean ± SD	p-value
0, 800	Normal Tissue	1.402 ± 0.20	>0.05
	Benign	0.708 ± 0.14	
	Malignant	0.524 ± 0.03	
0, 1000	Normal Tissue	1.606 ± 0.18	>0.05
	Benign	0.839 ± 0.15	
	Malignant	0.79 ± 0.014	
0, 1500	Normal Tissue	1.416 ± 0.15	<0.05
	Benign	0.665 ± 0.15	
	Malignant	0.52 ± 0.16	
0, 2000	Normal Tissue	1.25 ± 0.20	>0.05
	Benign	0.59 ± 0.19	
	Malignant	0.48 ± 0.09	

**Figure 1.**
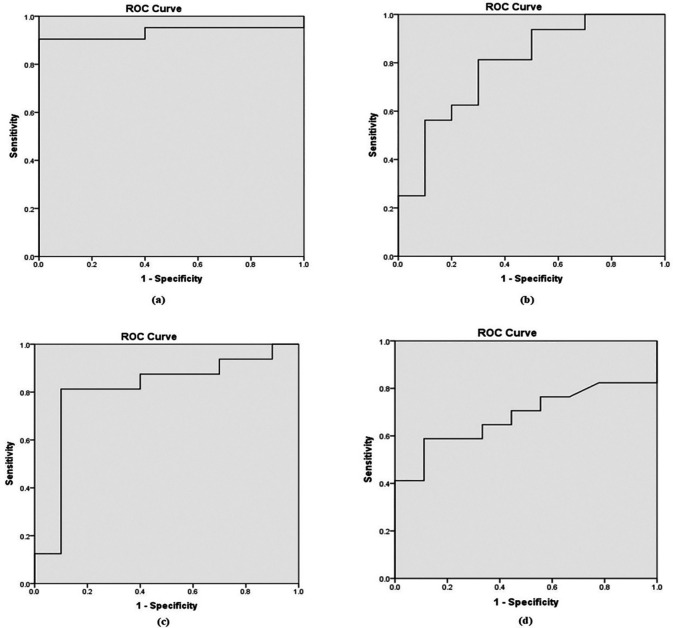
Receiver Operating Characteristic (ROC) curves derived from ADC values in differential diagnosis of benign from malignant lesions for b value of (a). 0,800 mm ^2^/s; (b). 0, 1000 mm ^2^/s; (c). 0, 1500 mm ^2^/s; and (d). 0, 2000 mm ^2^/s.

Based on the Receiver Operating Characteristic (ROC) curve ( Figure 1), the area under the curve (AUC) was 0.90, 0.71, 0.80, and 0.64 for b-values of 800, 1000, 1500, and 2000 mm²/s, respectively as shown in Table 5. To distinguish between benign and malignant prostate lesions, the AUC was significantly larger for b-values of 800 and 1500 mm ^2^/s. Additionally, the prime threshold points and the diagnostic power of each b value were ascertained by analyzing the ADC readings at various cut-off points.

**Table 5.** Cut off Threshold value, Sensitivity, Specificity at multiple b-value for distinguishing between Benign and Malignant Prostate Lesions at different b values.

b value (mm ^2^/s)	AUC	ADC- Cut Off X 10 ^–3^mm ^2^/s Mean SD	Sensitivity (%)	Specificity (%)
0, 800	0.90	0.481	90.9	83.3
0, 1000	0.71	0.510	90.9	79
0, 1500	0.80	0.389	94	87
0, 2000	0.64	0.351	77	88

## Discussion

MRI is an extensively accepted diagnostic tool for assessing prostate tissues and anomalies. DWI has a significant potential for assessing the structural properties of tissues and characterizing lesions. The effective diagnosis of a lesion requires its detection, and the b-value in DWI is a crucial factor in determining lesion conspicuity. In the present study, we observed that b-1500 mm ^2^/s had the highest SNR, SIR, and CNR in comparison to the other b values. Despite the intermediate kappa values across b-values, the high SNR, CNR, and SIR suggest that b-1500 mm ^2^/s has superior image quality, indicating that the sensitivity of the diffusion weighted imaging is heavily influenced by the b-value. Hence, selecting a smaller b-value results in considerable signal attenuation owing to the high mobility of water molecules. We observed that increasing the b-value decreased the SNR of the image, which is consistent with the findings of previous studies. ^12^

A recent study demonstrated the significance of b-values in the detection of prostate lesions in DWI sequences. We found that b-values of 0 and 1500 mm ^2^/s yielded optimal image quality. However, in the study reported by Kitajima et al., ^11^ noted that b1000 mm ^2^/s had a better SNR (48.7 **±**13.5 and 33.2 **±**7.9 for cancerous and non-cancerous tissue) and CNR of (15.6±8.1) compared to b2000 mm ^2^/s. In contrast, Nagayama et al. ^8^ determined that b800 offers a higher SNR and fewer artifacts when compared with higher b-values for the mapping of ADCs.

**Figure 2.**
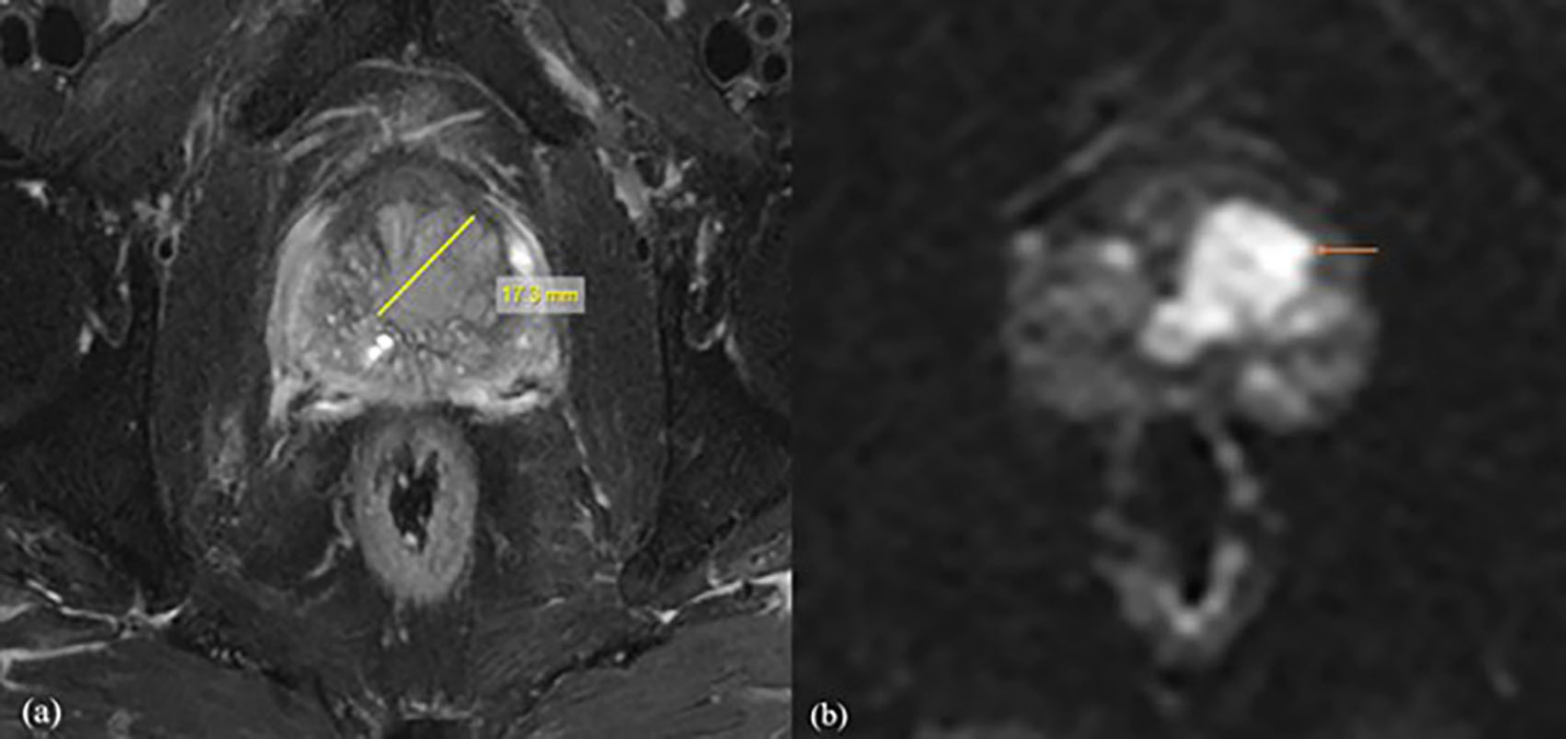
A 72-year-old male patient with difficulty urinating, the biochemistry revealed a PSA level of 58 mg/dl. Following a biopsy, the patient was diagnosed with prostatic acinar adenocarcinoma with a Gleason score of (4+3) = 7. For further investigation, the patient was referred for MRI Prostate. (a) Axial T2 fat suppression sequence showed a lesion with a length of 20.1mm, clearly delineating its boundaries. (b) The DWI with b value of 1500 mm ^2^/s demonstrated values of 0.511×10 ^-3^mm ^2^/s with decreased SNR and good CNR.

In the study by Rezaeian et al., ^12^ b 1200 mm ^2^/s was found to be an effective differential diagnosis technique owing to its low b-value, which allows DW images to show both extravascular molecular diffusion and perfusion characteristics, thus reducing the diagnostic accuracy of ADC values in distinguishing prostate cancer from healthy tissue. In a similar study, Rosenkranz et al. ^13^ indicated that prostate cancer diagnosis is most effective, with a b-value between 1500 and 2000 mm ^2^/s. In contrast, the higher b-values (3000-5000 mm ^2^/s) demonstrated inferior performance owing to inadequate or excessive signal suppression, resulting in poor anatomical clarity. According to Seung Soo Lee et al. ^14^ conducted a study comparing b1000 with b1800 and found that b1800 had improved accuracy and detection rates for lesions. In addition, we observed an increase in the accuracy rate for lesions classified as PI-RADS 4 or 5. As recommended by Chandarana et al., ^15^ multiparametric prostate MRI protocols should incorporate DWI sequences with b-values greater than 1000 mm ^2^/s for the effective differentiation of normal tissue from lesions, both benign and malignant, in which, according to our study, the optimal b-value is 0,1500 mm ^2^/sec. Based on the quantitative analysis of ADC values for b-values of 800, 1000, 1500, and 2000 mm ^2^/s obtained in the present study, it was shown that the b1500 ADC value is optimal for the differential diagnosis of prostate lesions ( Figure 2). For b value of 0,1500 mm ^2^/s, the mean ADC values were 1.416 ± 0.15 mm ^2^/s for normal tissue, 0.665 ± 0.15 mm ^2^/s for benign lesions, and 0.52 ± 0.16 mm ^2^/s for malignant lesions, with the threshold cut off ADC value of 0.389 × 10 ^−3^ mm ^2^/s, with of 94% sensitivity and 87%specificity. It was also found that for normal tissue, benign and malignant ADC values decreased with increasing b values, which may be due to perfusion or diffusion, as reported by Abbas Rezaeian et al. ^12^

The study by Amol Madanlal et al. ^13^ found that the with ADC values for malignant lesions were significantly lower (0.75 ± 0.19) compared to benign lesions (1.14 ± 0.14), with high sensitivity of 82.98%, specificity of 89.47%, and a positive predictive value of 95.12% in the differentiation between benign and malignant lesions with b value of 1000 mm ^2^/s. Kazuhiro Kitajima et al ^14^ reported an ADC cut-off value of 1.14 × 10 ^−3^ mm ^2^/s with a b-value of 1000 mm ^2^/s, in which malignant tissues exhibited significantly lower ADC values of 0.82 ± 0.27 mm ^2^/s compared to benign tissues with 1.69 ± 0.23 mm ^2^/s. Abbas Rezaeian et al, ^15^ reported the ADC for the malignant lesions to be 0.87 ±0.13 mm ^2^/s and for benign lesion 1.43±0.12 mm ^2^/s, with the ADC cut-off value of 0.94 × 10 ^−3^ mm ^2^/s at a b-value of 1200 mm ^2^/s, achieving 90.2% sensitivity, 92.6% specificity, and 91% overall accuracy indicating good diagnostic sequences for differential diagnosis of prostate lesions. Masako Nagayama et al, ^12^ reported mean ADC values of 1.00 ± 0.22 mm ^2^/s for malignant lesions and 1.56 ± 0.14 mm ^2^/s for benign lesions, with a threshold cutoff value of 1.35 × 10 ^−3^ mm ^2^/s, yielding sensitivity, specificity, and accuracy of 88%, 96%, and 93%, respectively. In addition, significant changes in ADC values may be attributed to necrosis or marked fibrosis, which may affect water diffusion or restriction. According to our analysis, there were slight differences in the threshold cut-off value in all previous studies, which could be attributed to different methods of calculating the qualitative ADC values, small sample sizes, and stages of cancer, in which more in-depth research needs to be conducted.

We observed that MRI strength did not influence ADC values in the differential diagnosis of prostate cancer. In contrast, a higher magnetic field strength of 3T provided better image quality owing to the improvements in the CNR and SNR. Significant changes in ADC values can occur because of necrosis or marked fibrosis, which affects water diffusion or restriction. The most notable benefit of DWI is that it can be easily integrated into screening protocols for high-risk populations, and when contrast-enhanced imaging is contraindicated, DWI is more reliable than T1- and T2-weighted imaging for the detection of prostate cancer. DWI can quantitatively characterize tumors (ADC value); therefore, it can be used as an alternative to invasive procedures, such as biopsies, which can cause incontinence, erectile dysfunction, infection, and septic shock.

This study has some limitations, including the small sample size and lack of an endorectal coil, which could have enhanced the image quality and prostate cancer localization using a dedicated coil. Future studies should increase sample sizes to improve the generalizability of findings and enhance statistical power. The utilization of an endorectal coil in MRI protocols may optimize the signal-to-noise ratio and spatial resolution, thereby improving the accuracy of prostate lesion detection. Furthermore, standardizing b-values across institutions and integrating multiparametric MRI with advanced machine learning algorithms could enhance diagnostic precision and support automated prostate cancer classification.

## Conclusion

Diffusion-weighted sequencing (DWI) in magnetic resonance imaging (MRI) is a valuable tool for both qualitative and quantitative evaluation of prostate pathology. According to our study, the optimal b-value for the detection and differential diagnosis of prostate lesions was 0,1500 mm ^2^/s. This sequence has the potential to enhance the positive predictive value of prostate cancer screening, and because it requires a short scan time and is highly sensitive, it can be used as a screening tool for high-risk populations. Standardization of b-value will allow for improved inter-study comparisons of the diagnostic accuracy of diffusion-weighted MR prostate imaging. Normalized ADC values can assist in the differential diagnosis of prostate lesions and tumors. Overall, DWI is a crucial sequence in multiparametric MRI (mpMRI) of the prostate, offering detailed information that enhances the accuracy of prostate cancer diagnosis and management.

## Ethics and consent

Ethical approval for this prospective study was obtained from the Institutional Ethics Committee (IEC 126/2023) of Kasturba Hospital, Manipal, India, on 4/05/2023. This study was registered in the Clinical Trial Registry of India (CTRI) and approval was received on the 09 ^th^ of June 2023, in which data collection was started on the 15 ^th^ of June 2023. All participants were fully informed about the study’s objectives and procedures. Written informed consent was obtained in compliance with the ethical principles set forth in the Declaration of Helsinki.

## Data Availability

Figshare: Annotated data set, https://doi.org/10.6084/m9.figshare.28219067.v2. ^16^ This project contains the following underlying data:
• The data consist of the qualitative and quantitative values of DWI Data are available under the terms of the Creative Commons Attribution 4.0 International license (CC-BY 4.0).

## References

[1] Laddha A Thomas A Nair DC : Outcomes of standard 12-core transrectal ultrasound-guided prostate biopsy in biopsy naive Indian men -single center experience. *Indian J. Urol.* 2020;36(3):179–183. 10.4103/iju.IJU_344_19 33082632 PMC7531374

[2] Tan CH Wang J Kundra V : Diffusion weighted imaging in prostate cancer. *Eur. Radiol.* 2011 Mar;21(3):593–603. 10.1007/s00330-010-1960-y 20936413

[3] Tamada T Ueda Y Ueno Y : Diffusion-weighted imaging in prostate cancer. *Magnetic Resonance Materials in Physics, Biology and Medicine.* 2022; Vol.35: p.533–547. Springer Science and Business Media Deutschland GmbH. 10.1007/s10334-021-00957-6

[4] Dkhar W Kadavigere R Mustaffa SP : Quantitative Evaluation for Differential Diagnosis of Breast Lesions in Diffusion-Weighted MR Imaging. *Health Technol (Berl).* 2021 Nov 1;11(6):1269–1275. 10.1007/s12553-021-00604-z

[5] Maurer MH Heverhagen JT : Diffusion weighted imaging of the prostate-principles, application, and advances. *Transl. Androl. Urol.* 2017;6:490–498. AME Publishing Company. 10.21037/tau.2017.05.06 28725591 PMC5503962

[6] Fennessy FM Maier SE : Quantitative diffusion MRI in prostate cancer: Image quality, what we can measure and how it improves clinical assessment. *Eur. J. Radiol.* 2023;167:111066. Elsevier Ireland Ltd. 10.1016/j.ejrad.2023.111066 37651828 PMC10623580

[7] Rezaeian A Ostovari M Hoseini-Ghahfarokhi M : Diffusion-weighted magnetic resonance imaging at 1.5 T for peripheral zone prostate cancer: the influence of the b-value combination on the diagnostic performance of apparent diffusion coefficient. *Pol. J. Radiol.* 2022 Jan 1;87:215–219. 10.5114/pjr.2022.115715 PMC9093206 35582606

[8] Nagayama M Watanabe Y Terai A : Determination of the cutoff level of apparent diffusion coefficient values for detection of prostate cancer. *Jpn. J. Radiol.* 2011 Aug;29(7):488–494. 10.1007/s11604-011-0586-6 21882091

[9] Dkhar W Kadavigere R Paruthikunnan SM : Image Quality Analysis for Optimization of b-value in Diffusion Weighted MRI of Breast. *Malays. J. Med. Health Sci.* 2020;16.

[10] IBM SPSS Statistics 28 Brief Guide This edition applies to version 28, release 0, modification 0 of IBM ^®^ SPSS ^®^ Statistics and to all subsequent releases and modifications until otherwise indicated in new editions.

[11] Chris ©, Reviewer K, Marshall E: community project encouraging academics to share statistics support resources All stcp resources are released under a Creative Commons licence. Reference Source

[12] Nagayama M Watanabe Y Terai A : Determination of the cutoff level of apparent diffusion coefficient values for detection of prostate cancer. *Jpn. J. Radiol.* 2011 Aug;29(7):488–494. 10.1007/s11604-011-0586-6 21882091

[13] Lahoti AM Lakhotiya AR Ingole SM : Role and application of diffusion-weighted imaging in evaluation of prostate cancer. *Indian J. Med. Paediatr. Oncol.* 2018 Jul 1;39(3):349–354. 10.4103/ijmpo.ijmpo_41_17

[14] Kitajima K Kaji Y Kuroda K : High b-value DiŠusion-weighted Imaging in Normal and Malignant Peripheral Zone Tissue of the Prostate: EŠect of Signal-to-Noise Ratio. *Magn. Reson. Med. Sci.* 2008;7:93–99. 10.2463/mrms.7.93 18603841

[15] Rezaeian A Ostovari M Hoseini-Ghahfarokhi M : Diffusion-weighted magnetic resonance imaging at 1.5 T for peripheral zone prostate cancer: the influence of the b-value combination on the diagnostic performance of apparent diffusion coefficient. *Pol. J. Radiol.* 2022 Jan 1;87:e215–e219. 10.5114/pjr.2022.115715 35582606 PMC9093206

[16] Dkhar W : Qualitative Data Quantitative Data.[Dataset]. *figshare.* 2025. 10.6084/m9.figshare.28219067.v2

