# Supplementary material for: Enhancing prostate cancer detection: The role of b-value and apparent diffusion coefficient in DWI
Source: F1000Res. 2025 Sep 19;14:155. Originally published 2025 Feb 4. [Version 4] doi: 10.12688/f1000research.161128.4 (PMC12504939; doi:10.12688/f1000research.161128.4)
Supplement: Peer review history

# Reviewer report: Reviewer response for version 4

**DOI:** 10.5256/f1000research.188480.r415648

**Published:** 2025-10-07

**Authors:** Jaseemudheen MM

**Affiliations:** 1 K.S. Hegde Medical Academy, NITTE, Mangalore, India

I appreciate the thorough and careful revisions made to the manuscript. The authors have responded to all queries with clarity and have thoughtfully addressed the concerns raised in the previous round of review. The removal of the CNR parameter has improved the consistency and readability of the results, and the additional explanations provided have enhanced the overall clarity of the manuscript. The study is well-structured, the methodology is sound, and the findings contribute valuable insights to the field. I am satisfied with the revisions and recommend the manuscript for indexing.

Is the work clearly and accurately presented and does it cite the current literature?

Yes

If applicable, is the statistical analysis and its interpretation appropriate?

Yes

Are all the source data underlying the results available to ensure full reproducibility?

Yes

Is the study design appropriate and is the work technically sound?

Yes

Are the conclusions drawn adequately supported by the results?

Yes

Are sufficient details of methods and analysis provided to allow replication by others?

Yes

Reviewer Expertise:

MRI, CT, Radiation safety

I confirm that I have read this submission and believe that I have an appropriate level of expertise to confirm that it is of an acceptable scientific standard.

# Reviewer report: Reviewer response for version 3

**DOI:** 10.5256/f1000research.187913.r413119

**Published:** 2025-09-19

**Authors:** Jaseemudheen MM

**Affiliations:** 1 K.S. Hegde Medical Academy, NITTE, Mangalore, India

I appreciate the thorough effort made in revising the manuscript and in addressing the earlier queries with clarity. The additional explanations have improved the overall readability of the work. However, regarding Table 3, the CNR values show pronounced skewness, with disproportionately large standard deviations in some cases exceeding the mean. This appears to result from combining data acquired on two different MRI machines, which may render the reported measure less meaningful in its current form. To avoid potential misinterpretation, you may consider either:

 (i) *removing this parameter from Table 3*, or

 (ii) *presenting the values separately for each scanner*, so that the variability is more transparently represented.

Is the work clearly and accurately presented and does it cite the current literature?

Yes

If applicable, is the statistical analysis and its interpretation appropriate?

Yes

Are all the source data underlying the results available to ensure full reproducibility?

Yes

Is the study design appropriate and is the work technically sound?

Yes

Are the conclusions drawn adequately supported by the results?

Yes

Are sufficient details of methods and analysis provided to allow replication by others?

Yes

Reviewer Expertise:

MRI, CT, Radiation safety

I confirm that I have read this submission and believe that I have an appropriate level of expertise to confirm that it is of an acceptable scientific standard, however I have significant reservations, as outlined above.

# Reviewer report: Reviewer response for version 2

**DOI:** 10.5256/f1000research.178777.r408413

**Published:** 2025-09-08

**Authors:** Jaseemudheen MM

**Affiliations:** 1 K.S. Hegde Medical Academy, NITTE, Mangalore, India

This is a well-conducted and clearly written study addressing an important aspect of prostate MRI optimization. The authors have presented their methods and results in detail, and the findings contribute valuable insights into the role of b-values and ADC in differentiating prostate lesions. The discussion is comprehensive and relates well to prior literature

1. Section- MRI Technique: Presenting scanner details (Separately for both scanners), in a clear protocol table, will improve readability.
2. Methods: Clarify whether all patients were imaged on both scanners (Philips 1.5T and United Imaging 3T) or divided between them, since field strength could influence results (Not ADC but other factors).
3. Consistently expand abbreviations at first mention (e.g., SI- Signal Intensity, NEX- Number of Excitations).
4. In **Table 2**, the reported CNR values show disproportionately large standard deviations, in some cases exceeding the mean, which raises concerns about calculation accuracy or data consistency. Please recheck the CNR computations and clarify whether these represent SD or another measure of variability (e.g., variance). Consider alternative representation (e.g., median ± IQR) if the data are highly skewed.
5. In **Table 3**, while mean ADC values are presented for normal, benign, and malignant tissues across different b-values, a pairwise comparison (e.g., benign vs malignant, malignant vs normal, benign vs normal- In an additional Table) would strengthen the statistical analysis and clarify which differences are statistically significant. Please consider including appropriate pairwise comparison tests with correction for multiple testing (post-hoc pairwise tests)

Is the work clearly and accurately presented and does it cite the current literature?

Yes

If applicable, is the statistical analysis and its interpretation appropriate?

Yes

Are all the source data underlying the results available to ensure full reproducibility?

Yes

Is the study design appropriate and is the work technically sound?

Yes

Are the conclusions drawn adequately supported by the results?

Yes

Are sufficient details of methods and analysis provided to allow replication by others?

Yes

Reviewer Expertise:

MRI, CT, Radiation safety

I confirm that I have read this submission and believe that I have an appropriate level of expertise to confirm that it is of an acceptable scientific standard, however I have significant reservations, as outlined above.

# Response

**Published:** 2025-09-08

**Authors:** Winniecia Dkhar

**Affiliations:** Medical Imaging Technology, Manipal Acdemy of Higher Education, Manipal, Karnataka, India

1) Reviewer Comments:- Section- MRI Technique: Presenting scanner details (Separately for both scanners), in a clear protocol table, will improve readability.

 Author Response:- We thank the reviewer for this suggestion. The MRI Technique section has been revised, and the scanner and sequence details are now presented in **Table 1** for clarity and readability.

 2) Reviewer Comments:- Methods: Clarify whether all patients were imaged on both scanners (Philips 1.5T and United Imaging 3T) or divided between them, since field strength could influence results (Not ADC but other factors).

 Author Response:- We thank the reviewer for this valuable comment. We have clarified this point in the revised Methods section. Patients were imaged on either the Philips Achieva 1.5T or the United Imaging uMR 3.0T scanner, depending on availability. Each patient underwent MRI on a single scanner only, and no patient was imaged on both systems.

 3) Reviewer Comments:- Consistently expand abbreviations at first mention (e.g., SI- Signal Intensity, NEX- Number of Excitations).

 Author Response:- We thank the reviewer for this observation. All abbreviations have now been expanded at their first mention in the manuscript.

 4) Reviewer Comments:- In **Table 2**, the reported CNR values show disproportionately large standard deviations, in some cases exceeding the mean, which raises concerns about calculation accuracy or data consistency. Please recheck the CNR computations and clarify whether these represent SD or another measure of variability (e.g., variance). Consider alternative representation (e.g., median ± IQR) if the data are highly skewed.

 Author Response:- We thank the reviewer for this comment. All values in Table 2, including SIR, SNR, and CNR, were calculated as **mean ± standard deviation (SD)**. The data were not skewed, and no alternative representation was necessary.

 5) Reviewer Comments:-  In **Table 3**, while mean ADC values are presented for normal, benign, and malignant tissues across different b-values, a pairwise comparison (e.g., benign vs malignant, malignant vs normal, benign vs normal- In an additional Table) would strengthen the statistical analysis and clarify which differences are statistically significant. Please consider including appropriate pairwise comparison tests with correction for multiple testing (post-hoc pairwise tests)

 Author Response:- We thank the reviewer for this suggestion. In this study, the primary objective was to report **mean ADC values and ranges** for normal, benign, and malignant prostate tissues at multiple b-values. Due to the relatively small sample size in the benign group performing pairwise statistical comparisons may not provide robust or reliable results. Therefore, we have reported the overall trends without post-hoc pairwise analysis, which we believe is appropriate given the exploratory nature of this study.

# Reviewer report: Reviewer response for version 2

**DOI:** 10.5256/f1000research.178777.r369839

**Published:** 2025-04-28

**Authors:** Manna Debnath

**Affiliations:** 1 Bapubhai Desaibhai Patel Institute of Paramedical Sciences, Charotar University of Science and Technology, Changa, Gujarat, India

The authors have carefully reviewed and corrected the manuscript based on the suggestions provided. All the corrections made by the authors are satisfactory. The manuscript is now ready for indexing.

Is the work clearly and accurately presented and does it cite the current literature?

Yes

If applicable, is the statistical analysis and its interpretation appropriate?

Yes

Are all the source data underlying the results available to ensure full reproducibility?

Yes

Is the study design appropriate and is the work technically sound?

Yes

Are the conclusions drawn adequately supported by the results?

Yes

Are sufficient details of methods and analysis provided to allow replication by others?

Yes

Reviewer Expertise:

Medical Imaging Technology_MRI

I confirm that I have read this submission and believe that I have an appropriate level of expertise to confirm that it is of an acceptable scientific standard.

# Reviewer report: Reviewer response for version 1

**DOI:** 10.5256/f1000research.177120.r365942

**Published:** 2025-02-26

**Authors:** Manna Debnath

**Affiliations:** 1 Bapubhai Desaibhai Patel Institute of Paramedical Sciences, Charotar University of Science and Technology, Changa, Gujarat, India

1. The abstract is well written, highlighting this research article's overall summary.

 2. Is there any Indian study published on prostate cancer detection using DWI? If so, please cite the study in the Introduction section and provide a detailed overview of the Indian perspective.

 3. Page no. 3, in the MRI techniques section, 2 ^nd^ last line, please correct the sentence as with a combination of **five** b-values (b=0, 800,1000,1500 and 2000 mm2/s).

 4. Page no. 3, in the Image analysis section, 2 ^nd^ paragraph, expand SI. Most probably it is Signal Intensity.

 5. On page 4, in the Results section, first paragraph, the sentence 'The mean ADC values for benign lesions were 0.708 × 0.149 mm²/s, 0.839 × 0.15 mm²/s, 0.665 × 0.15 mm²/s, and 0.59 × 0.19 mm²/s for the corresponding b-values' should be corrected to use the '±' sign instead of '×', as the author has reported mean and standard deviation (SD). Additionally, in the b = 800 mm²/s value, the SD is written as 0.149 in the text but 0.14 in Table 3. Please ensure uniform decimal formatting throughout the paper to avoid reader confusion.

 6. In the very next line of page 4, in the Results section, the sentence 'In a similar manner, the mean ADC values of malignant lesions were 0.4949 mm²/s, 0.014 mm²/s, 0.52 mm²/s, and 0.48 mm²/s, respectively.' contains discrepancies between the data reported in the text and the values presented in Table 3. Please rectify these inconsistencies to ensure accuracy.

 7. The discussion and conclusion are well written. However, I suggest adding 2–3 lines on future recommendations that could guide further research on prostate imaging using MRI.

Is the work clearly and accurately presented and does it cite the current literature?

Yes

If applicable, is the statistical analysis and its interpretation appropriate?

Yes

Are all the source data underlying the results available to ensure full reproducibility?

Yes

Is the study design appropriate and is the work technically sound?

Yes

Are the conclusions drawn adequately supported by the results?

Yes

Are sufficient details of methods and analysis provided to allow replication by others?

Yes

Reviewer Expertise:

Medical Imaging Technology_MRI

I confirm that I have read this submission and believe that I have an appropriate level of expertise to confirm that it is of an acceptable scientific standard.
